# Temporal and disproportionality analysis of vaccine-associated Guillain-Barré syndrome: a 21-year VAERS study

**DOI:** 10.3389/fneur.2026.1814946

**Published:** 2026-08-04

**Authors:** Yonglong Su, Yirong Huang, Lili Pan, Honghong Fu, Xun Wang

**Affiliations:** 1 Department of Clinical Pharmacy, Xiamen Haicang Hospital, Xiamen, Fujian, China; 2 Department of Pharmacy, Fujian Medical University Union Hospital, Fuzhou, Fujian, China; 3 Fujian Institute of Hematology, Fujian Provincial Key Laboratory on Hematology, Fujian Medical University Union Hospital, Fuzhou, Fujian, China; 4 Department of Pharmacology and Toxicology, Fujian Institute for Food and Drug Quality Control, Fuzhou, Fujian, China; 5 Department of Proctology, Fujian Provincial People’s Hospital, Fuzhou, Fujian, China

**Keywords:** disproportionality analysis, Guillain-Barré syndrome, pharmacovigilance, signal detection, vaccine safety

## Abstract

**Background:**

Guillain-Barré syndrome (GBS) remains a critical vaccine safety concern. We conducted a comprehensive pharmacovigilance analysis of 21-year VAERS data to identify vaccine-GBS disproportionality signals and temporal patterns.

**Methods:**

We analyzed 9,755,435 VAERS reports (2004–2024) including 2,216 GBS cases using four disproportionality algorithms (ROR, PRR, IC, EBGM). Time-to-onset was compared between 1,534 GBS and 120,763 non-GBS events using Kaplan–Meier analysis.

**Results:**

Twenty-six vaccines met all four algorithm criteria, predominantly influenza formulations (19/26; RORs 2.4–8.5), validating historical surveillance observations. Novel signals emerged for both RSV vaccines (Arexvy ROR 3.01, Abrysvo ROR 6.82) and zoster vaccine (ROR 3.01), consistent with post-marketing estimates of 3–9 excess cases per million doses. COVID-19 vaccines showed no concordant signals despite established Janssen associations, reflecting denominator dilution from 7.89 million COVID-19 reports (81% of the database). GBS exhibited characteristic delayed onset—median 13 days (IQR 5–29) versus 0 days for non-GBS events (*p* < 0.001)—with 44.8% of cases occurring 8–30 days post-vaccination, the biologically plausible window for vaccine-triggered autoimmunity. Demographically, GBS reports showed male predominance (OR 2.4) and were overwhelmingly classified as serious outcomes compared with non-GBS reports (76.1% vs. 9.6%, *p* < 0.001); serious outcomes included death, life-threatening events, hospitalization (new or prolonged), or permanent disability.

**Conclusion:**

Multiple vaccines showed disproportionate GBS reporting signals with delayed temporal patterns consistent with biological plausibility; because these derive from passive surveillance, they are hypothesis-generating and do not establish causality. Despite rare associations, absolute risks (1–9 per million doses) are far outweighed by disease prevention benefits, supporting current vaccination policies with ongoing surveillance.

## Introduction

Guillain-Barré syndrome (GBS) is an acute immune-mediated polyradiculoneuropathy that causes rapidly progressive ascending weakness and may progress to respiratory failure and paralysis (1, 2). With an annual incidence of approximately 10–20 cases per million people worldwide, GBS remains uncommon but clinically severe, frequently requiring hospitalization and, in a subset of patients, intensive care support (1–4). The condition has long been recognized as a rare adverse event following a range of antecedent infections and, less commonly, immunizations, consistent with an immune-triggered pathogenesis (1, 2).

The vaccine-GBS association emerged during the 1976 influenza vaccination campaign, during which an excess risk of approximately 1 case per 100,000 vaccine recipients was observed (5, 6). Subsequent studies have found much smaller but still detectable GBS risks with seasonal influenza vaccines, on the order of 1–2 excess cases per million doses (7–9). More recently, post-licensure surveillance has identified GBS associations with newly approved vaccines, including the recombinant zoster vaccine (Shingrix), with approximately a few excess cases per million doses, and respiratory syncytial virus (RSV) vaccines, with approximately 7–9 excess cases per million doses (10, 11). In addition, U. S. safety monitoring in July 2021 documented elevated GBS reporting following the Janssen COVID-19 vaccine, estimated at approximately 7–8 cases per million doses (12, 13).

Spontaneous reporting systems such as the Vaccine Adverse Event Reporting System (VAERS) serve as critical early-warning tools for detecting potential vaccine–adverse event associations and generating hypotheses for further evaluation (14–16). Because passive surveillance systems generally lack active denominators and are subject to reporting variability, disproportionality analysis has become a standard approach for quantitative signal detection in spontaneous report databases (17, 18). Four major algorithms are commonly employed: the Reporting Odds Ratio (ROR) and Proportional Reporting Ratio (PRR), which assess relative reporting frequency using frequentist measures (19, 20); the Information Component (IC), a Bayesian metric used in WHO-led pharmacovigilance systems to quantify deviation from expected reporting (21); and the Empirical Bayesian Geometric Mean (EBGM), used in FDA data-mining frameworks and incorporating Bayesian shrinkage to stabilize estimates under sparse data conditions (22, 23). Using multiple concordant algorithms improves robustness, as agreement across methods with different statistical assumptions helps reduce false-positive findings attributable to chance, small counts, or database subset effects (17, 18).

A critical aspect of GBS surveillance is temporal pattern analysis. Unlike immediate vaccine reactions, GBS typically emerges days to weeks after an immune trigger, consistent with post-infectious immunological mechanisms (1, 2). Most vaccine-associated GBS cases occur within 6 weeks post-vaccination, often peaking around 2–3 weeks (24, 25). Characterizing time-to-onset patterns compared with other adverse events strengthens biological plausibility and helps distinguish true safety signals from background or reporting noise (24, 25).

In this study, we present a comprehensive 21-year analysis of GBS reports in VAERS from 2004 to 2024. We applied four signal detection algorithms to identify vaccines with disproportionate GBS reporting and examined temporal onset patterns. By integrating multiple analytic approaches across this extended timeframe, we aimed to clarify the scope of GBS reporting in VAERS, identify which vaccines demonstrate the strongest disproportionality signals, characterize when cases tend to occur, and interpret these findings in the context of known risks and inherent limitations of passive surveillance.

## Methods

### Data source and study design

We conducted a comprehensive pharmacovigilance analysis using the U. S. Vaccine Adverse Event Reporting System (VAERS), a national passive surveillance database co-managed by the Centers for Disease Control and Prevention (CDC) and the U. S. Food and Drug Administration (FDA) (14, 15). We extracted all VAERS reports with vaccination dates from January 1, 2004 through December 31, 2024, encompassing 9,755,435 total adverse event reports.

### Case definition and classification

Guillain-Barré syndrome (GBS) cases were identified using the Medical Dictionary for Regulatory Activities (MedDRA) Preferred Term “Guillain-Barré syndrome” (PT code: 10018767). Reports containing this MedDRA term were classified as GBS cases (26). For disproportionality analyses, we constructed a vaccine-specific 2 × 2 table for each vaccine and applied four signal-detection algorithms—Reporting Odds Ratio (ROR), Proportional Reporting Ratio (PRR), Empirical Bayesian Geometric Mean (EBGM), and Information Component (IC)—using pre-specified thresholds (19–23). Vaccines meeting the positivity criteria across all four algorithms were defined as concordant (four-method) signals. These 26 concordant vaccines were carried forward for downstream characterization and ordered by the number of GBS reports attributed to each vaccine for presentation purposes only; across these 26 vaccines, a total of 2,216 GBS reports were identified. Key variables extracted included patient demographics (age, sex), vaccine type administered, serious outcome classification (death, life-threatening illness, hospitalization, disability), vaccination date, and adverse event onset date. Reports were restricted to single-vaccine exposure, defined as reports in which only one vaccine product was recorded. Reports listing co-administration of two or more vaccines were excluded so that each report, and therefore each reported GBS event, could be analytically linked to a single vaccine product in the disproportionality analyses. This linkage was used for analytic classification and does not imply causal attribution.

#### Inclusion and exclusion criteria

We included VAERS reports received between January 1, 2004 and December 31, 2024. The date window was applied using the report receipt date (RECVDATE) rather than the vaccination date, because RECVDATE is non-missing for all reports and unambiguously defines the reporting window, whereas the vaccination date is missing or imprecise for a proportion of reports. Reports were then restricted to single-vaccine exposure, defined as reports in which only one vaccine product was recorded; reports listing co-administration of two or more vaccine products were excluded. No restriction by U. S. state or country of origin was applied; both domestic and non-domestic VAERS records were therefore retained.

#### Signal-detection background

For the signal-detection stage, all eligible single-vaccine reports served as the background population. At the report level, the source VAERS dataset contained 2,665,843 reports. After excluding 174,769 reports outside the report-receipt date window and 229,368 reports involving more than one vaccine exposure, 2,261,706 eligible single-vaccine reports remained. Disproportionality analyses (ROR, PRR, IC, and EBGM) were performed at the PT-record level. Because a single report may contain multiple adverse-event PTs, these eligible single-vaccine reports corresponded to 9,755,435 PT-level records, which formed the signal-detection background; this full background, rather than the final 26-vaccine dataset, was used for all disproportionality estimation.

#### Downstream analytic dataset

For downstream characterization, we retained vaccine products that showed concordant positive signals across all four algorithms, yielding 26 vaccine products and 160,394 reports (2,216 reports coded with the MedDRA preferred term “Guillain-Barré syndrome” and 158,178 non-GBS reports). Thus, the reduction from the full signal-detection background to the downstream analytic dataset reflected the prespecified focus on vaccine products with four-algorithm concordant GBS signals, rather than exclusion due to missing demographic or outcome data. Missing age, sex, and outcome values were retained and categorized as “Not available” or unknown. Time-to-onset analyses were restricted to reports with a non-missing, non-negative interval between vaccination and symptom onset (122,297 reports; 1,534 GBS and 120,763 non-GBS).

### Disproportionality signal detection

We applied four established signal–detection algorithms to identify vaccine-GBS associations with disproportionate reporting patterns. Each algorithm evaluates whether GBS is reported more frequently than expected for a given vaccine relative to the VAERS background (17, 18). For each vaccine, we constructed a 2 × 2 contingency table in which a denotes the number of GBS reports for the target vaccine, b the number of non-GBS reports for the target vaccine, c the number of GBS reports for all other vaccines, and d the number of non-GBS reports for all other vaccines.

### We applied four algorithms with the following criteria

*Reporting Odds Ratio (ROR)*: ROR = (a × d)/(b × c). A signal was defined as a lower 95% confidence interval (CI) > 1.0 with ≥3 reports (19).

*Proportional Reporting Ratio (PRR)*: PRR = [a/(a + b)]/[c/(c + d)]. A signal was defined as PRR ≥ 2.0, χ^2^ ≥ 4.0, and ≥3 reports (20).

*Information Component (IC)*: A Bayesian metric quantifying deviation from expected reporting; a signal was defined as IC025 (lower 95% credibility limit) > 0 (21).

*Empirical Bayesian Geometric Mean (EBGM)*: The FDA’s Multi-Item Gamma Poisson Shrinker framework with Bayesian shrinkage; a signal was defined as EBGM05 (5th percentile) > 2.0 (22, 23).

Complete equations and parameter settings are provided in Supplementary Table 1. A concordant positive signal was defined as meeting all four algorithm thresholds simultaneously. We selected this stringent criterion to prioritize specificity by requiring agreement across methods with different statistical assumptions and shrinkage behaviors, reducing spurious signals from sparse counts or subset effects (17, 18).

### Demographic and clinical characteristics of GBS reports

We compared demographic and clinical characteristics between GBS and non-GBS reports using chi-square tests for categorical variables. Age was stratified into clinically relevant groups (<18, 18–49, 50–64, ≥65 years, and not available). The 50-year cut point reflects the recommended age threshold for recombinant zoster vaccination, whereas the ≥65-year category was retained as a conventional older-adult threshold in descriptive epidemiology and vaccine safety analyses. This grouping also allowed the sparse former ≥85-year stratum to be combined with the 65–84-year group to improve estimate stability. Sex distribution and serious outcome proportions were compared between groups. Statistical significance was set at *p* < 0.05 (two-tailed). All analyses were performed using R version 4.3.1.

### Time-to-onset analysis

For reports with complete date information, we calculated the interval (in days) from vaccination to symptom onset. We categorized onset timing into 10 predefined intervals: 0 days (vaccination day), 1–7, 8–30, 31–60, 61–90, 91–120, 121–150, 151–180, 181–360, and >360 days. These cutoffs capture immediate reactions, the early post-vaccination period, the peak GBS risk window, an extended surveillance period, and longer-term intervals.

We constructed Kaplan–Meier curves to visualize cumulative incidence of adverse event onset over time. The log-rank test was used to compare distributions between GBS and non-GBS groups. Median time-to-onset and interquartile ranges (IQR) were calculated for each group. For vaccine-specific analyses, we summarized median onset times and IQRs for the 26 concordant vaccines.

### Temporal trend analysis

To evaluate year-by-year reporting patterns, we calculated annual GBS reporting proportion as: (GBS cases per year/total VAERS reports per year) × 100%. We examined trends across four epidemiological periods defined by major public health events: pre-H1N1 pandemic (2004–2008), H1N1 pandemic era (2009–2010), post-pandemic normalization (2011–2020), and COVID-19 vaccination era (2021–2024).

## Results

### Overview of VAERS GBS reports (2004–2024)

Between January 2004 and December 2024, VAERS received 9,755,435 total adverse event reports following vaccination. After applying predefined inclusion/exclusion and data-completeness criteria for disproportionality analyses, the analytic dataset comprised 160,394 reports, including 2,216 reports coded with PT = GBS and 158,178 non-GBS reports.

### Temporal trends in GBS reporting

**Figure 1:**
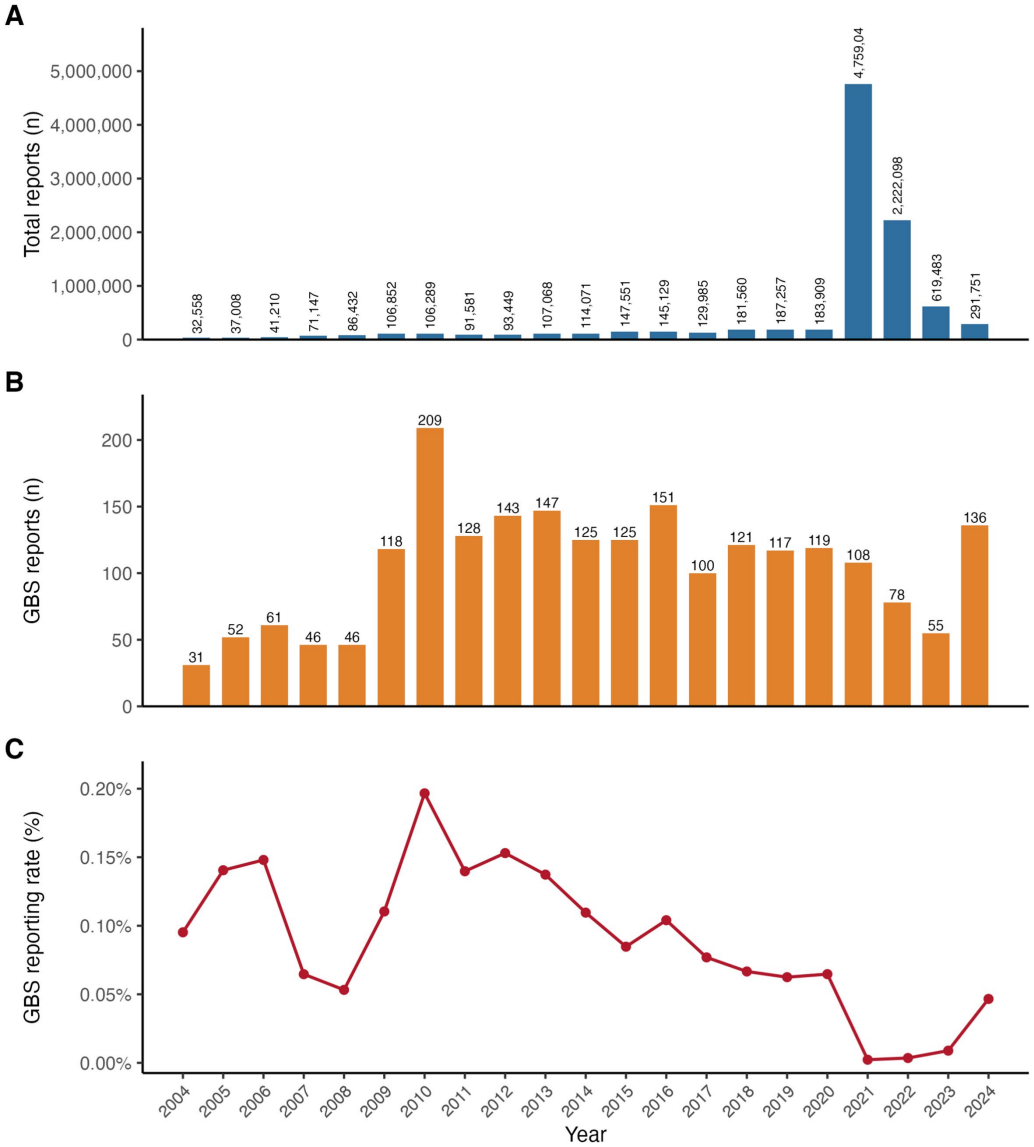
Temporal trends in VAERS total reports and Guillain-Barré syndrome (GBS) reports with the annual GBS reporting rate (2004–2024). The figure is split into three panels that share a common year axis. **(A)** The total number of VAERS reports received each year (blue bars). **(B)** The annual number of reports coded with the MedDRA preferred term “Guillain-Barré syndrome” (orange bars; the count is labeled above each bar). **(C)** The annual GBS reporting rate, calculated as GBS reports/total reports × 100. Across 2004–2024, the database comprised 9,755,435 total reports and 2,216 GBS reports. Reporting volume peaked in 2021–2022 during the COVID-19 vaccination period, accompanied by a marked decline in the GBS reporting rate over the same years, consistent with a denominator-expansion effect in passive surveillance rather than a direct inference of reduced risk. GBS, Guillain-Barré syndrome; MedDRA, Medical Dictionary for Regulatory Activities; PT, preferred term; VAERS, Vaccine Adverse Event Reporting System.

Annual VAERS reporting volume and the proportion of reports coded with PT = GBS varied over time (Figure 1). The full 2004–2024 dataset comprised 9,755,435 total VAERS reports and 2,216 GBS-coded reports.

### Three distinct temporal patterns emerged

*Pre-COVID Era (2004–2020)*: Total reports increased from 32,558 (2004) to 183,909 (2020). Across this period, annual GBS reporting rates generally ranged from ~0.05% to ~0.20%, with a clear elevation during the 2009–2010 H1N1 pandemic vaccination period (peak 0.1966% in 2010; 209 GBS reports).

*COVID-19 Vaccination Era (2021–2022)*: Total reporting surged to 4,759,047 (2021) and 2,222,098 (2022), representing 81% of the entire 21-year database. Despite this unprecedented reporting volume, GBS rates appeared to decline precipitously (0.0023 and 0.0035% respectively). This reduction reflects a denominator effect rather than true risk decrease—absolute GBS case counts remained stable (110 cases in 2021, 78 in 2022, median 94/year for 2021–2024), while the massive influx of COVID-19 vaccine adverse event reports diluted the proportional GBS rate.

Partial Normalization (2023–2024): As COVID-19 vaccination transitioned to routine immunization schedules, reporting volumes decreased (139,433 reports in 2023; 291,751 in 2024) and GBS rates partially recovered (0.0466% in 2024), approaching but not reaching pre-pandemic levels.

These temporal patterns validate the necessity of disproportionality analysis over simple rate calculations, as raw GBS proportions can be misleading when denominator reporting intensity varies dramatically across years.

### Demographic and clinical characteristics of GBS reports

GBS reports exhibited distinct demographic profiles compared with non-GBS adverse events (Table 1). All between-group comparisons were statistically significant (*p* < 0.001).

**Table 1:** Baseline characteristics of VAERS reports with and without Guillain-Barré syndrome (2004–2024).

Characteristics	Non-GBS (*n* = 158,178)	GBS (*n* = 2,216)	Total (*n* = 160,394)
Age group, *n* (%)***			
< 18 years	25,370 (16.0)	138 (6.2)	25,508 (15.9)
18–49 years	44,059 (27.9)	494 (22.3)	44,553 (27.8)
50–64 years	27,977 (17.7)	500 (22.6)	28,477 (17.8)
≥ 65 years	30,126 (19.0)	559 (25.2)	30,685 (19.1)
Not available	30,646 (19.4)	525 (23.7)	31,171 (19.4)
Sex, *n* (%)***			
Female	95,361 (60.3)	953 (43.0)	96,314 (60.0)
Male	45,305 (28.6)	1,097 (49.5)	46,402 (28.9)
Unknown	17,512 (11.1)	166 (7.5)	17,678 (11.0)
Serious outcome, *n* (%)***			
No	143,006 (90.4)	530 (23.9)	143,536 (89.5)
Yes	15,172 (9.6)	1,686 (76.1)	16,858 (10.5)

VAERS, Vaccine Adverse Event Reporting System; GBS, Guillain-Barré syndrome. ***P < 0.001 by chi-square test comparing GBS versus non-GBS groups. Serious outcomes include death, life-threatening events, hospitalization (new or prolonged), or permanent disability. Data are presented as n (%) unless otherwise indicated.

Age distribution. GBS reports were skewed toward older adults, with a clear age gradient. Pediatric reports (<18 years) were underrepresented among GBS cases (6.2% vs. 16.0% of non-GBS reports), as were adults aged 18–49 years (22.3% vs. 27.9%). In contrast, GBS reports were progressively enriched at older ages, from 50 to 64 years (22.6% vs. 17.7%) to ≥65 years (25.2% vs. 19.0%; all *p* < 0.001). Age was missing in 23.7% of GBS reports and 19.4% of non-GBS reports.

Sex distribution. A male predominance was observed in GBS reports. Males represented 49.5% of GBS cases compared with 28.6% of non-GBS reports (*p* < 0.001), corresponding to an unadjusted odds ratio of approximately 2.4 for male sex and GBS classification. Females comprised 43.0% of GBS reports versus 60.3% of non-GBS reports. Unknown sex was recorded in 7.5% of GBS reports and 11.1% of non-GBS reports.

Outcome severity. GBS reports were predominantly serious. Overall, 76.1% (1,686/2,216) of GBS reports met FDA serious outcome criteria (death, life-threatening illness, hospitalization or prolongation of hospitalization, or permanent disability), compared with 9.6% (15,172/158,178) of non-GBS reports (*p* < 0.001).

### Vaccines with concordant GBS disproportionality signals

**Figure 2:**
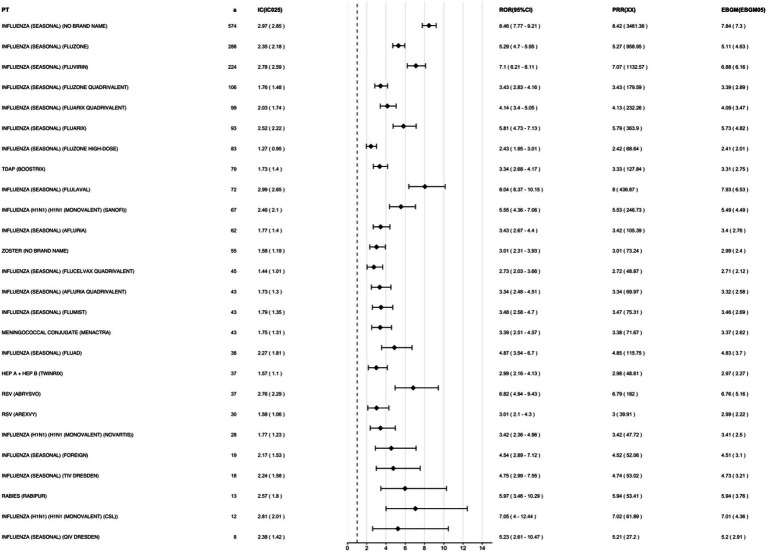
Disproportionality signal metrics for the 26 vaccine-GBS pairs in VAERS (2004–2024). This forest plot displays, for each vaccine identified with a four-method positive signal, the reporting odds ratio (*ROR*) with 95% confidence interval. Vaccines are listed by name (including product or brand in parentheses) and ordered by descending number of GBS reports. All displayed *RORs* have lower confidence bounds >1.0, indicating a significant GBS signal. For context, the lower panel also shows each vaccine’s Information Component lower bound (*IC0_25_*) and EBGM percentile (*EBGM_05_*) in parentheses. All values exceed their signal thresholds (*IC0_25_*0, EBGM_05_> 2). Influenza vaccines (multiple formulations and brands) comprise the majority of signals, consistent with an accumulation of GBS reports historically associated with flu vaccination. Other notable signals include the zoster vaccine, meningococcal conjugate (Menactra), rabies (Rabipur), RSV vaccines (Abrysvo and Arexvy), and the combination hepatitis A/B vaccine (Twinrix). These results highlight which vaccines disproportionately appear in GBS reports, but do not imply causation without further evidence.

Among all vaccine products in VAERS, 26 vaccine types met all four signal-detection thresholds simultaneously and were ranked by the number of PT = GBS reports attributed to each vaccine. The corresponding disproportionality estimates are summarized in Figure 2 and Supplementary Table 3.

### Influenza vaccines

A clear pattern was the predominance of influenza vaccine formulations, which accounted for 19 of the 26 concordant four-method signals. Seasonal influenza vaccines consistently showed the highest disproportionality. The largest signal was observed for influenza vaccine (seasonal), unspecified formulation (574 GBS reports; ROR 8.46, 95% CI 7.77–9.21; IC 2.97, IC025 2.85; EBGM 7.84, EBGM05 7.30). Several licensed seasonal products also met concordant signal criteria, including Fluzone (288 reports; ROR 5.29, 95% CI 4.70–5.95), Fluvirin (224 reports; ROR 7.10, 95% CI 6.21–8.11), Fluzone High-Dose (83 reports; ROR 2.43, 95% CI 1.95–3.01), and Flulaval (72 reports; ROR 8.04, 95% CI 6.37–10.15). Quadrivalent influenza products were also represented among concordant signals, including Fluzone Quadrivalent (106 reports; ROR 3.43), Fluarix Quadrivalent (99 reports; ROR 4.14), Flucelvax Quadrivalent (45 reports; ROR 2.73), and Afluria Quadrivalent (43 reports; ROR 3.48).

Pandemic-era 2009 H1N1 monovalent vaccines appeared as distinct concordant signals, including H1N1 monovalent (Sanofi) (67 reports; ROR 2.99, 95% CI 2.65–3.38), H1N1 monovalent (Novartis) (28 reports; ROR 3.42, 95% CI 2.36–4.96), and H1N1 monovalent (CSL) (12 reports; ROR 7.02, 95% CI 4.00–12.44).

### Zoster vaccines

Herpes zoster vaccines (unspecified brand) also met the four-method concordant criteria, with 55 PT = GBS reports and elevated disproportionality estimates (ROR 3.01, 95% CI 2.31–3.93; IC025 1.19; EBGM05 2.40) (Figure 2). In a sensitivity analysis restricted to reports received after the November 2020 U. S. discontinuation of the live zoster vaccine (RECVDATE, 1 November 2020–31 December 2024), the zoster–GBS signal remained positive across all four algorithms, with a higher reporting odds ratio than in the full-period analysis (ROR 4.63, 95% CI 3.54–6.06; PRR 4.62, χ^2^ = 151.44; EBGM 4.58, EBGM05 3.66; IC 2.19, IC025 1.80; *n* = 54).

### Meningococcal conjugate vaccine (MenACWY)

Menactra (MenACWY) met the four-method concordant criteria, with 43 PT = GBS reports and elevated disproportionality estimates (ROR 3.39, 95% CI 2.51–4.57) (Figure 2).

### Tetanus-diphtheria-acellular pertussis vaccine

Tdap (Boostrix) showed 79 GBS reports (ROR 3.34, 2.68–4.17), a novel finding requiring further investigation given inconsistent historical evidence for tetanus toxoid-GBS associations.

### Respiratory syncytial virus vaccines

Both recently approved RSV vaccines for older adults met the four-method concordant criteria despite relatively short time on the market. Arexvy (GSK) had 30 PT = GBS reports (ROR 3.01, 95% CI 2.10–4.30; EBGM05 2.22), and Abrysvo (Pfizer) had 37 reports with higher disproportionality (ROR 6.82, 95% CI 4.94–9.43; EBGM05 5.16) (Figure 2).

### Hepatitis A + B combination vaccine

Twinrix showed 37 GBS reports (ROR 2.99, 2.16–4.13), representing a hypothesis-generating finding without established epidemiologic confirmation.

### Rabies vaccine

Rabipur (13 reports; ROR 5.97, 3.46–10.29) exhibited high disproportionality, though absolute case counts remain small. The life-saving importance of rabies post-exposure prophylaxis requires balanced risk–benefit assessment.

Additional vaccines with concordant signals included multiple seasonal influenza formulations (Fluarix, Afluria, Flumist, Fluad) and several foreign/unspecified influenza products.

### COVID-19 vaccines and limitations of concordant signal detection

COVID-19 vaccines were notably absent from the list of 26 four-method concordant signals (Supplementary Table 2). For mRNA vaccines (Pfizer-BioNTech and Moderna), disproportionality estimates remained consistently below the pre-specified thresholds across algorithms (e.g., ROR < 1.0, IC025 < 0, EBGM05 < 2), indicating no disproportionate reporting of PT = GBS relative to the very large volume of non-GBS COVID-19 vaccine reports in VAERS during 2021–2024.

For the adenoviral vector vaccine (Janssen/Johnson and Johnson), VAERS-based disproportionality showed partial concordance: the ROR exceeded unity (ROR 1.66, 95% CI 1.52–1.82) and the Bayesian IC criterion was met (IC025 > 0), but PRR and EBGM criteria were not simultaneously satisfied (Supplementary Table 2). This pattern is compatible with a “denominator dilution” effect in passive surveillance, in which exceptionally large volumes of reports across diverse adverse events can expand vaccine-specific comparator strata and attenuate relative disproportionality metrics even when an elevated risk has been identified in active surveillance systems. Importantly, the absence of COVID-19 vaccines from the four-method concordant list should not be interpreted as evidence of no GBS risk. Rather, it indicates that under our stringent requirement for cross-method agreement, COVID-19 vaccines did not emerge among the most disproportionately represented vaccine categories for PT = GBS in VAERS over 2004–2024. This interpretation is consistent with U. S. safety monitoring and active surveillance findings documenting an elevated GBS risk following Janssen vaccination in 2021, while generally not observing comparable signals for mRNA vaccines (12, 13).

### Time-to-onset analysis: distinct temporal signature of vaccine-associated GBS

Of 2,216 GBS reports, 1,534 (69.2%) had complete vaccination and onset dates for temporal analysis; all percentages in this section use this evaluable for time to onset denominator (*n* = 1,534).

**Figure 3:**
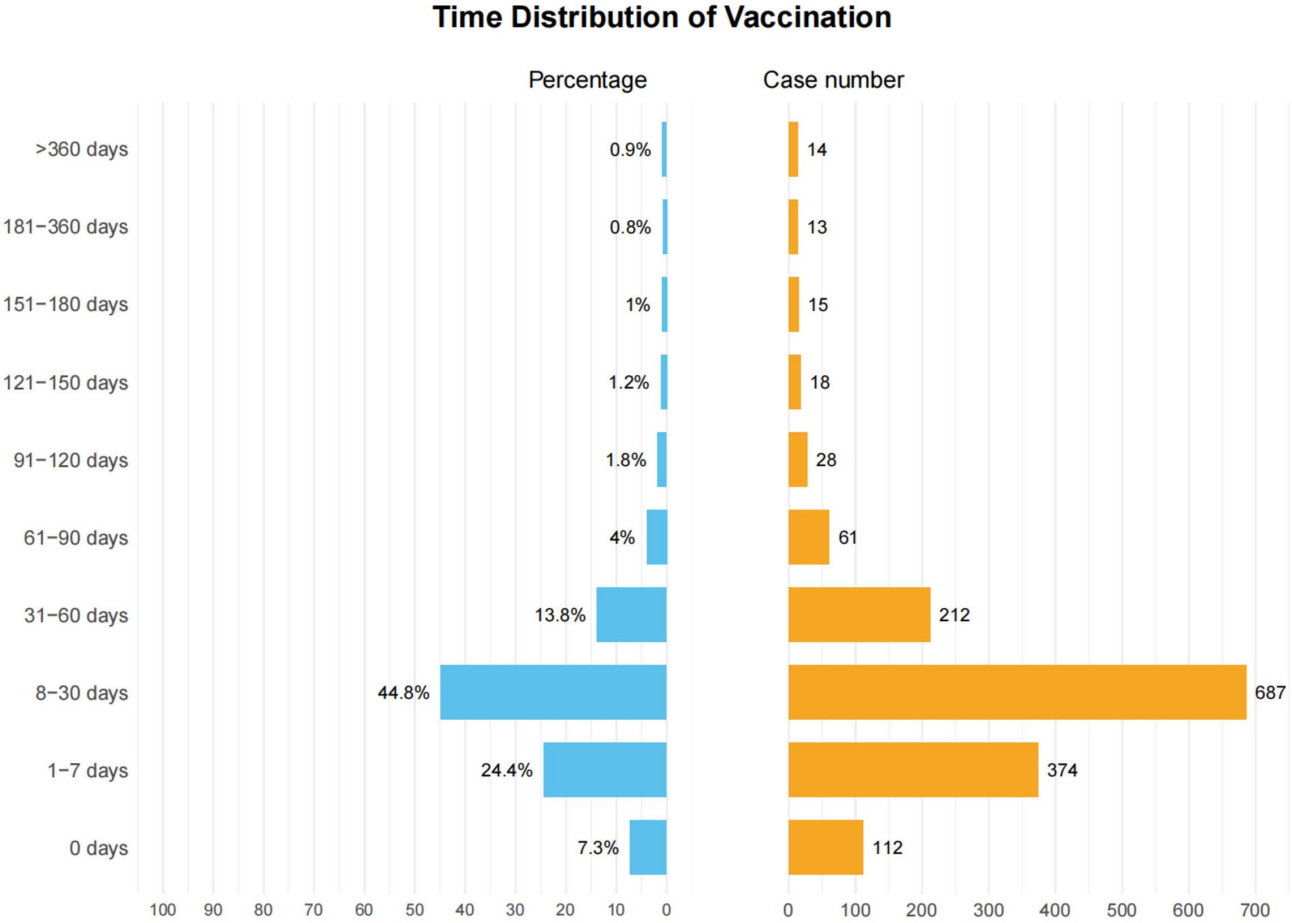
Time distribution from vaccination to GBS onset in VAERS (2004–2024). This figure summarizes the onset interval (days) between vaccination and reported Guillain-Barré syndrome (GBS) symptom onset among cases with a recorded onset date (*n* = 1,534). The left panel shows the proportion of cases in each prespecified time window, and the right panel shows the corresponding case counts. Most reports occurred within 30 days after vaccination, peaking at 8–30 days (44.8%, 687/1,534) followed by 1–7 days (24.4%, 374/1,534) and 31–60 days (13.8%, 212/1,534). Smaller proportions were observed beyond 60 days, including 61–90 days (4.0%, 61), 91–120 days (1.8%, 28), 121–150 days (1.2%, 18), 151–180 days (1.0%, 15), 181–360 days (0.8%, 13), and >360 days (0.9%, 14). The “0 days” category indicates same-day onset (7.3%, 112/1,534).

Onset Interval Distribution (Figure 3). The modal onset window was 8–30 days post-vaccination, accounting for 687 cases (44.8% of total). Combining the first month (0–30 days) captured 1,173 cases (76.5%), with the following distribution: day 0, 112 (7.3%); days 1–7, 374 (24.4%); days 8–30, 687 (44.8%); days 31–60, 212 (13.8%); days 61–90, 61 (4.0%); and >90 days, 88 (5.7%). This temporal clustering is compatible with an immune-mediated latency, as GBS typically develops days to weeks after an immunologic trigger (1, 2, 25). The small day-0 component likely reflects background coincidence, imprecise dating, or documentation practices rather than an immediate vaccine-triggered onset.

Extended window summaries showed 1,385 cases (90.3%) within 60 days; 1,446 cases (94.3%) within 90 days; 1,507 cases (98.2%) within 180 days, which is broadly consistent with the commonly used 42-day (6-week) surveillance window applied in vaccine safety monitoring for GBS (27).

**Figure 4:**
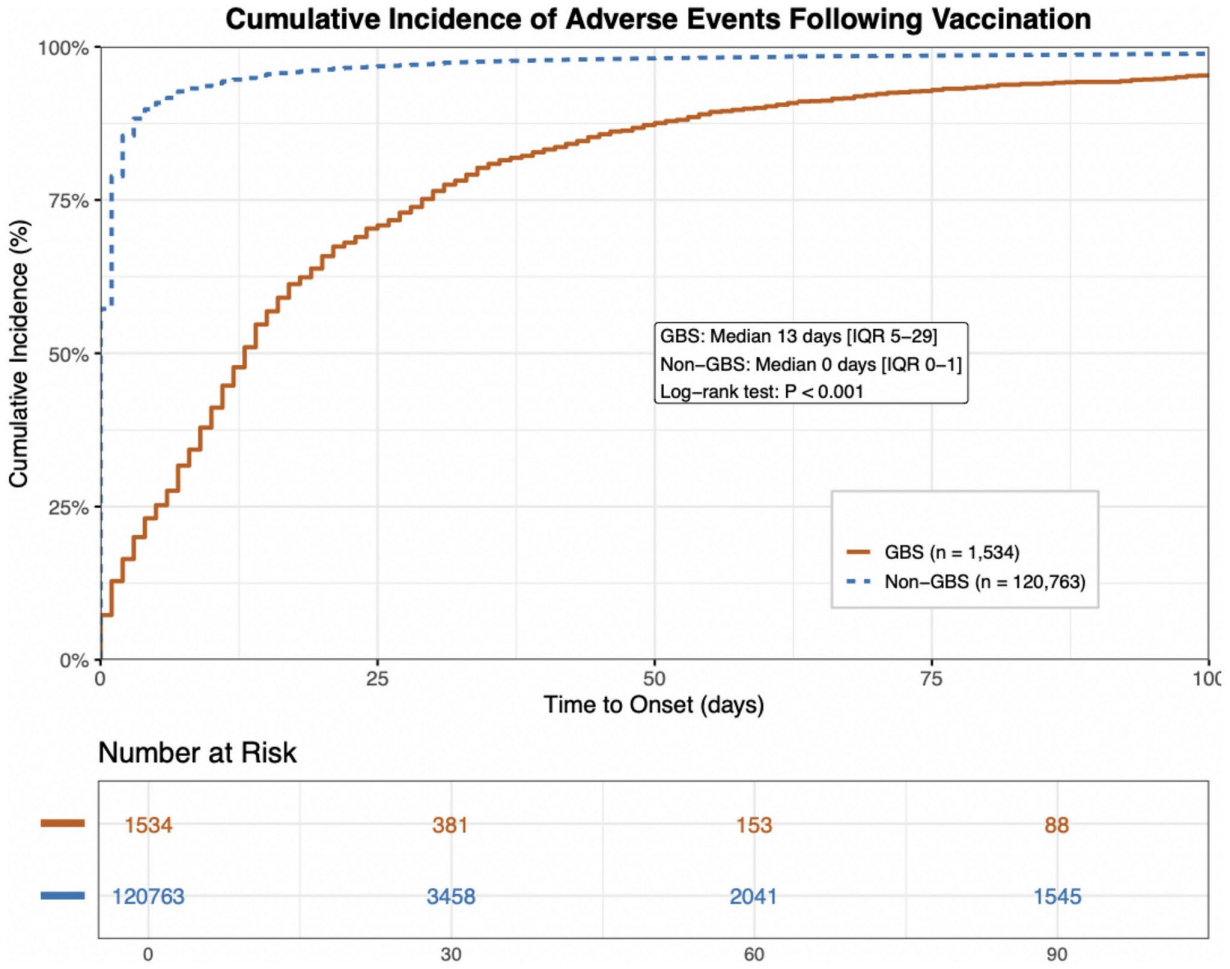
Cumulative incidence of reported onset following vaccination for GBS versus non-GBS events in VAERS (2004–2024). Cumulative incidence curves compare time from vaccination to reported symptom onset among GBS cases (*n* = 1,534) and non-GBS reports with a recorded onset date (*n* = 120,763), truncated at 100 days. GBS reports showed a delayed onset distribution (median 13 days, IQR 5–29) compared with non-GBS reports (median 0 days, IQR 0–1). Curves were compared using the log-rank test (*p* < 0.001). Numbers at risk at 0, 30, 60, and 90 days are displayed below the plot.

Comparative analysis with non-GBS events (Figures 3, 4). Among 120,763 non-GBS reports with complete dates, onset was concentrated at very short intervals (approximately 60% on day 0 and ~31% within days 1–7, with <10% beyond day 7) (Figure 3). Cumulative incidence curves differed significantly between GBS and non-GBS reports (log-rank *p* < 0.001) (Figure 4). Median time-to-onset was 13 days (IQR 5–29) for GBS versus 0 days (IQR 0–1 day) for non-GBS reports, indicating substantially delayed onset for GBS relative to typical vaccine adverse events. By day 7 post-vaccination, the vast majority of non-GBS events had occurred, whereas only about one-third of GBS onsets were observed; conversely, most GBS onsets accrued over the first 2–8 weeks.

**Figure 5:**
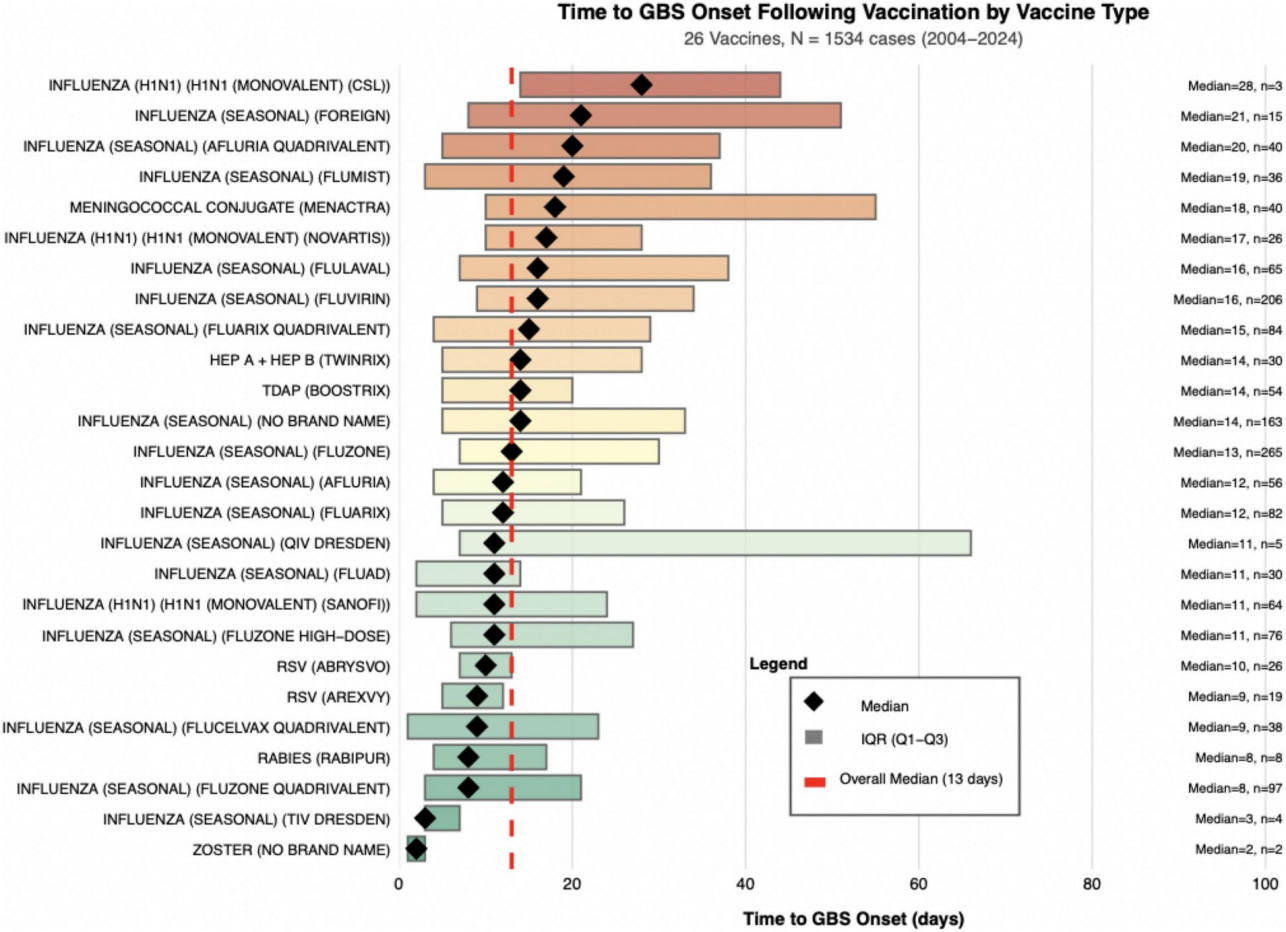
Time to Guillain-Barré Syndrome onset following vaccination by vaccine type. Horizontal bar chart showing median time-to-onset (black diamonds) and IQR (gray bars, Q1–Q3) for 26 vaccines, sorted by descending median (*n* = 1,534 total cases). Red dashed line indicates overall median of 13 days. Median onset ranges from 2 days (Zoster) to 28 days (Influenza H1N1 CSL), with most vaccines clustering in 10–20 day window. This delayed pattern distinguishes GBS from immediate vaccine reactions and aligns with immune-mediated pathophysiology requiring 1–4 weeks for anti-ganglioside antibody generation. Influenza vaccines show heterogeneous timing (11–28 days), RSV vaccines show early onset (9–10 days), and Menactra demonstrates delayed presentation (19 days). Sample sizes vary (*n* = 2–265), affecting estimate precision. Abbreviations: GBS, Guillain-Barré syndrome; IQR, interquartile range; Q1, 25th percentile; Q3, 75th percentile.

Vaccine-specific temporal patterns (Figure 5). Across the 26 vaccines with concordant four-method signals, median time to GBS onset varied by vaccine type among reports with available onset intervals, ranging from approximately 2–28 days (Figure 5). Despite vaccine-specific variation, interquartile ranges generally spanned the ~5–30 day window, reinforcing that GBS, regardless of vaccine trigger category, tends to exhibit delayed post-immunization onset.

## Discussion

In this 21-year VAERS analysis (2004–2024), we identified vaccines with concordant disproportionality signals for PT = GBS using a stringent four-algorithm framework and observed a delayed onset profile that contrasted with the immediate onset pattern typical of many other reported adverse events (14–16). We identified three key findings. First, 26 vaccine types met concordant positivity across all four methods, with influenza vaccines comprising the majority (19/26). Second, concordant signals were also detected for recently introduced vaccines, including RSV and zoster vaccines, consistent with recent post-licensure safety attention (10, 11). Third, among time-to-onset–evaluable GBS reports, onset peaked at 8–30 days (44.8%), whereas non-GBS events clustered predominantly on day 0. Although passive surveillance cannot establish causality, the observed latency pattern is compatible with the expected clinical course of an immune-mediated neuropathy and provides supportive temporal context (1, 2, 25).

Influenza vaccines warrant particular attention given their widespread use. Across influenza formulations, disproportionality estimates showed several-fold over-representation of GBS reports relative to the VAERS background. ROR ranged from 2.4 to 8.5 across products, with IC values broadly between 1 and 3. These measures should be interpreted as disproportionate reporting rather than absolute risk, because VAERS lacks an active denominator and is influenced by reporting dynamics (14–18). Nonetheless, the dominance of influenza vaccines in the concordant signal set is broadly consistent with prior epidemiologic evidence suggesting that any increased GBS risk following seasonal influenza vaccination—when observed—is small (on the order of ~1–2 excess cases per million doses) and occurs within a several-week post-vaccination window (5–9, 24). Overall, these findings support continued product-level monitoring and transparent risk communication, while emphasizing the need for analytic designs to quantify risk precisely and to contextualize signals within broader vaccine benefit–risk considerations (14–18).

### Novel signals: RSV and zoster vaccines in the post-approval window

Both FDA-approved RSV vaccines showed concordant signals (Arexvy: ROR 3.01; Abrysvo: ROR 6.82), demonstrating that stringent multi-algorithm frameworks can detect emerging safety patterns in early post-licensure periods. As expected for a passive system, these VAERS-based findings are hypothesis-generating rather than risk estimates; however, their direction is broadly consistent with regulatory safety communications indicating an elevated GBS reporting signal following RSV vaccination in older adults (estimated at ~7–9 excess cases per million doses in the post-authorization analysis that informed labeling updates), supporting continued product-level monitoring as real-world exposure accumulates (11). Similarly, we observed a signal for zoster vaccine reports coded in VAERS without brand specification (ROR 3.01; 55 reports). Although this passive-surveillance signal cannot be attributed to a specific zoster vaccine product, its direction is consistent with post-marketing evidence from U. S. Medicare data suggesting a small excess risk of GBS following recombinant zoster vaccination, which has informed risk communication and labeling updates (10). Because VAERS does not distinguish recombinant (RZV/Shingrix) from live (Zostavax) product, we cannot directly attribute our signal to a specific formulation. However, in a sensitivity analysis restricted to the period after the November 2020 discontinuation of live zoster vaccine in the United States, when zoster vaccination was predominantly, although not exclusively, RZV, the signal persisted and strengthened (ROR 4.63, 95% CI 3.54–6.06; *n* = 54). This finding is consistent with, though not proof of, a contribution from the recombinant vaccine. Because this restriction was applied by report receipt date (RECVDATE) rather than vaccination date, reports received after November 2020 may still include earlier live-vaccine (Zostavax) exposures; residual live-vaccine misclassification therefore cannot be excluded and represents a limitation of this analysis. While recombinant zoster vaccine is an adjuvanted formulation designed to elicit strong immune responses in older adults, the biologic basis for rare GBS reports after vaccination remains uncertain and cannot be inferred from passive surveillance data alone (28). Only two zoster–GBS reports had calculable non-negative onset intervals (0 and 5 days; median, 2.5 days; range, 0–5 days). Therefore, the apparent proximity to day 0 should be interpreted as a consequence of data sparsity rather than evidence of an established short-latency pattern. From a clinical perspective, these findings reinforce pragmatic considerations for both vaccine classes: (1) maintaining vigilance for progressive, ascending weakness during the conventional post-vaccination monitoring window; (2) incorporating rare GBS reporting patterns into shared decision-making while contextualizing them against substantial disease burden—RSV is associated with approximately 60,000–160,000 hospitalizations and 6,000–10,000 deaths annually among U. S. adults aged ≥65 years (29), and herpes zoster affects roughly one-third of individuals over their lifetime with a meaningful proportion developing postherpetic neuralgia (30); and (3) continued reporting of suspected cases to strengthen post-marketing signal evaluation and facilitate more definitive risk quantification in active-surveillance or cohort-based designs (14–16).

### The COVID-19 vaccine paradox: interpreting absence of concordant signals

The absence of COVID-19 vaccines from our 26 concordant four-method signals should be interpreted cautiously. This pattern seems inconsistent with U. S. safety monitoring that identified an elevated GBS risk following the Janssen (Ad26. COV2. S) vaccine in 2021, while not identifying a comparable signal for mRNA products (12, 13). In our dataset, COVID-19 vaccines accounted for an exceptionally large share of reports during 2021–2024 (7.89 million; 81% of the 2004–2024 database), which substantially expands the vaccine-specific “denominator” of non-GBS adverse events and can attenuate disproportionality metrics even when absolute GBS counts are non-trivial. Consistent with this, Janssen demonstrated partial evidence of disproportionate reporting (ROR 1.66, 95% CI 1.52–1.82; IC025 0.21) but did not meet PRR and EBGM thresholds simultaneously, and therefore did not qualify as a four-method concordant signal under our pre-specified criteria. More broadly, this illustrates a practical limitation of disproportionality approaches: signal detection reflects not only event frequency but also background reporting intensity, product-specific reporting behavior, and the aggregation of reports across heterogeneous time periods and vaccine categories. Accordingly, during mass vaccination campaigns, passive surveillance signals are best interpreted alongside controlled epidemiologic designs and active surveillance systems that can quantify risk with explicit denominators and defined risk windows (13, 31, 32).

### Temporal evidence supporting biological plausibility

A key finding is the clear separation in our study between the onset-time distributions for GBS and non-GBS reports (median 13 vs. 0 days; log-rank *p* < 0.001). This latency pattern is compatible with the clinical course of an immune-mediated neuropathy, in which symptoms typically develop days to weeks after an immunologic trigger rather than immediately after exposure (1, 2, 25). In our VAERS onset-evaluable subset, GBS reports concentrated in the 8–30 day window (44.8%), whereas non-GBS adverse events clustered predominantly on day 0, consistent with the timing of many common post-vaccination reactions. While passive surveillance data cannot establish causality, the enrichment of GBS onset in weeks 2–4 provides temporal coherence that is aligned with established immunologic concepts in GBS (including molecular mimicry and anti-ganglioside antibody responses described in infection-associated disease) (25, 27, 29). In addition, the finding that the vast majority of GBS onsets occurred within 90 days (94.3%) and 180 days (98.2%) supports the practical use of predefined post-vaccination monitoring windows (often centered on ~6 weeks) for GBS safety surveillance in clinical and post-marketing settings (24, 27). Notably, a small fraction of reports recorded onset on the vaccination day (7.3%), which likely reflects pre-existing or prodromal symptoms, coincident presentation at the time of vaccination, or date-recording imprecision rather than immediate vaccine-triggered neurologic injury, and this should be interpreted accordingly in passive reporting data.

### Demographic risk factors and clinical phenotype

Our demographic and clinical patterns—male predominance (OR ~2.4), skewing toward older age groups, and a high proportion classified as serious (76%)—are broadly consistent with established GBS epidemiology and the expected clinical severity of the syndrome, supporting the interpretation that VAERS reports coded as PT = GBS largely reflect clinically meaningful events rather than random misclassification (1–4, 25). The male predominance observed here parallels prior epidemiologic summaries reporting higher incidence in males, although the mechanisms underlying sex differences in GBS remain incompletely understood and should not be inferred from VAERS data alone (3, 33). The older age distribution is also directionally consistent with increasing baseline susceptibility to GBS with age and with the fact that several vaccines contributing prominent signals in our analysis (e.g., influenza, zoster, RSV) are preferentially administered to older adults (3, 4, 11, 34).

The markedly higher “serious outcome” proportion among GBS reports compared with non-GBS reports (76% vs. 9%) has two practical implications. First, it aligns with the recognized clinical course of GBS, for which hospitalization is common and a subset of patients require intensive monitoring and supportive therapies (including immunomodulatory treatment and, in severe cases, ventilatory support) (1, 2, 25).

Second, it is compatible with known reporting behavior in passive surveillance systems, where severe events are more likely to be reported than mild, self-limited reactions; therefore, VAERS may preferentially capture a larger share of clinically severe GBS presentations than of less serious adverse events overall (14, 16, 35).

### Study strengths and limitations

This study has several strengths. First, the 21-year observation window (2004–2024) captures long-term secular patterns as well as periods of intense reporting variability linked to major public health events. Second, we used a stringent definition of a “concordant signal” that required positivity across four commonly used disproportionality frameworks, which increases specificity relative to any single-method approach. Third, we complemented disproportionality findings with time-to-onset profiling and direct comparison against non-GBS reports, providing an internal temporal benchmark. Finally, several signals identified in our VAERS analysis were directionally consistent with published post-licensure epidemiologic evidence for selected vaccines, offering contextual support for interpretation (10, 11, 14, 17).

Important limitations should be acknowledged. VAERS is a passive, voluntary reporting system and is subject to underreporting, variable report completeness, and reporting stimulation, particularly during periods of heightened public attention such as the COVID-19 vaccination campaign (14–16). VAERS data also do not permit clinical verification of diagnoses, standardized case adjudication, or comprehensive adjustment for confounding (e.g., age, baseline risk, comorbidity, and vaccine-target population differences), and therefore cannot establish incidence or causality (14–16). Disproportionality analyses quantify disproportionate reporting relative to the database background rather than absolute risk, and signals should be interpreted as hypothesis-generating and best followed by analytic designs with explicit denominators and predefined risk windows (17, 18).

Finally, we applied established signal thresholds (ROR 95% CI lower bound >1; PRR ≥ 2 with χ^2^ ≥ 4; IC025 > 0; EBGM05 > 2) without formal multiplicity adjustment. Although requiring four-method concordance provides some protection against false positives—particularly for sparse strata—chance findings remain possible for vaccines with smaller counts or heterogeneous reporting patterns, underscoring the need for replication and, where feasible, active-surveillance confirmation (18, 36).

### Public health implications and future directions

Our findings support three immediate applications. First, continued GBS surveillance for established signal settings (influenza, zoster, RSV) with product-specific attention as formulations and target populations evolve. Second, hypothesis generation for less-studied vaccine products (e.g., Tdap, combination hepatitis, rabies vaccines) that merit evaluation using controlled epidemiologic designs. The historical meningococcal conjugate vaccine (Menactra) signal similarly reflects focused post-marketing evaluation in the mid-2000s, with any attributable GBS risk—if present—considered very small (on the order of <1 excess case per million doses) (37). In particular, the rabies signal warrants caution: these reports were coded to Rabipur, the ex-U. S. trade name of the vaccine licensed domestically as RabAvert, and derive largely from manufacturer-forwarded, non-U. S. reports; together with the small case count, this finding is best regarded as hypothesis-generating. Third, risk communication frameworks that transparently acknowledge rare GBS reporting signals while contextualizing absolute risk magnitude and disease-prevention benefits. Importantly, these results do not warrant changes to current vaccination recommendations. Consistent with ACIP guidance (38), a history of GBS within 6 weeks after a prior dose of the same vaccine is generally considered a precaution rather than an absolute contraindication, and decisions should be individualized in the context of disease risk and expected vaccine benefit.

Future research should pursue several directions: (1) application of advanced data-mining approaches (e.g., tree-based scan statistics) that can account for multiple testing across hierarchical diagnosis groupings and help detect unexpected event patterns under heterogeneous reporting environments (39); (2) integration of VAERS hypothesis-generating signals with active surveillance platforms (e.g., VSD/claims-based systems) using self-controlled designs to quantify attributable risk while controlling for time-invariant confounding (36); and (3) targeted clinical and laboratory characterization of suspected vaccine-associated GBS cases to improve case validation, phenotype definition, and mechanistic inference.

## Conclusion

In this 21-year VAERS analysis, a stringent four-algorithm framework identified a set of vaccine types with concordant disproportionality signals for PT = GBS and a delayed onset profile among time-to-onset–evaluable reports. These observations are best interpreted as signals that can prioritize further evaluation rather than as direct estimates of risk. Ongoing pharmacovigilance remains essential to detect product-specific variations, temporal clusters, or emergent patterns as vaccine use changes, while transparent communication should place rare neurologic reports in their appropriate epidemiologic context to sustain public confidence and guide clinically meaningful vigilance.

## Data Availability

The datasets presented in this study can be found in online repositories. The names of the repository/repositories and accession number(s) can be found at: Vaccine Adverse Event Reporting System (VAERS) database (https://vaers.hhs.gov/).
