# Supplementary material for: Temporal and disproportionality analysis of vaccine-associated Guillain-Barré syndrome: a 21-year VAERS study
Source: Front Neurol. 2026 Aug 4;17:1814946. doi: 10.3389/fneur.2026.1814946 (PMC13481260; doi:10.3389/fneur.2026.1814946)
Supplement: Supplementary file 2 [file Supplementary_file_2.docx]

**Supplementary Table 2. Signal detection results for Guillain-Barré syndrome (GBS) by COVID-19 vaccine type using four major algorithms (VAERS 2004–2024).**

| Vaccine  (Platform ) | GBS cases (n) | ROR  (95% CI) | PRR (χ²) | EBGM (EBGM05) | IC (IC025) |
| --- | --- | --- | --- | --- | --- |
| Pfizer-BioNTech (mRNA) | 2,345 | 0.53  (0.50–0.56) | 0.53  (665.52) | 0.68 (  0.65) | −0.56 (−0.63) |
| Moderna (mRNA) | 643 | 0.32  (0.30–0.35) | 0.32  (840.09) | 0.38  (0.36) | −1.39 (−1.51) |
| Janssen (Adenoviral) | 539 | 1.66*  (1.52–1.82) | 1.66  (132.03) | 1.61  (1.50) | 0.69*  (0.56) |
| Pfizer Bivalent (mRNA) | 34 | 0.54  (0.38–0.75) | 0.54  (13.66) | 0.54  (0.41) | −0.90 (−1.38) |
| COVID19 (UNKNOWN) | 30 | 2.34*  (1.63–3.35) | 2.34*  (22.85) | 2.33  (1.73) | 1.22*  (0.70) |
| Moderna Bivalent (mRNA) | 7 | 0.14  (0.07–0.30) | 0.14  (36.29) | 0.14  (0.08) | −2.81 (−3.83) |

Concordant 4-algorithm signals: 0 of 6 products

Pre-specified signal thresholds (consistent with the primary analysis):

* ROR positive if the lower bound of the 95% CI > 1 and case count ≥ 3.

*PRR positive if PRR ≥ 2, χ² ≥ 4, and case count ≥ 3.

* EBGM positive if EBGM05 > 2.

* IC positive if IC025 > 0.

Interpretation: Despite established epidemiologic evidence for Janssen-GBS association (~7 cases per million doses), no COVID-19 vaccines achieved four-algorithm concordance. This reflects denominator dilution from unprecedented reporting volumes which attenuates relative disproportionality metrics. Janssen showed partial signals (ROR and IC positive) but failed PRR and EBGM thresholds. This discordance illustrates VAERS

methodologic limitations during mass vaccination campaigns rather than absence of true risk.

Abbreviations: CI, confidence interval; EBGM, Empirical Bayesian Geometric Mean;

EBGM_05_, 5th percentile; GBS, Guillain-Barré syndrome; IC, Information Component;

IC_025,_ lower 95% credibility limit; mRNA, messenger RNA; PRR, Proportional

Reporting Ratio; ROR, Reporting Odds Ratio; VAERS, Vaccine Adverse Event Reporting

System; χ², chi-squared statistic.
