# Supplementary material for: Temporal and disproportionality analysis of vaccine-associated Guillain-Barré syndrome: a 21-year VAERS study
Source: Front Neurol. 2026 Aug 4;17:1814946. doi: 10.3389/fneur.2026.1814946 (PMC13481260; doi:10.3389/fneur.2026.1814946)
Supplement: Supplementary file 3 [file Supplementary_file_3.docx]

**Supplementary Table 3. Disproportionality signal metrics for the 26 vaccines with concordant four-algorithm GBS signals in VAERS (2004–2024).**

| **Vaccine (product/brand)** | **GBS reports (n)** | **ROR (95% CI)** | **PRR (χ²)** | **EBGM (EBGM_05_)** | **IC (IC_025_)** |
| --- | --- | --- | --- | --- | --- |
| **Influenza, seasonal (unspecified formulation)** | **574** | **8.46 (7.77–9.21)** | **8.42 (3461.38)** | **7.84 (7.30)** | **2.97 (2.85)** |
| **Influenza, seasonal (Fluzone)** | **288** | **5.29 (4.70–5.95)** | **5.27 (958.95)** | **5.11 (4.63)** | **2.35 (2.18)** |
| **Influenza, seasonal (Fluvirin)** | **224** | **7.10 (6.21–8.11)** | **7.07 (1132.57)** | **6.88 (6.16)** | **2.78 (2.59)** |
| **Influenza, seasonal (Fluzone Quadrivalent)** | **106** | **3.43 (2.83–4.16)** | **3.43 (179.59)** | **3.39 (2.89)** | **1.76 (1.48)** |
| **Influenza, seasonal (Fluarix Quadrivalent)** | **99** | **4.14 (3.40–5.05)** | **4.13 (232.26)** | **4.09 (3.47)** | **2.03 (1.74)** |
| **Influenza, seasonal (Fluarix)** | **93** | **5.81 (4.73–7.13)** | **5.79 (363.90)** | **5.73 (4.82)** | **2.52 (2.22)** |
| **Influenza, seasonal (Fluzone High-Dose)** | **83** | **2.43 (1.95–3.01)** | **2.42 (68.64)** | **2.41 (2.01)** | **1.27 (0.95)** |
| **Tdap (Boostrix)** | **79** | **3.34 (2.68–4.17)** | **3.33 (127.84)** | **3.31 (2.75)** | **1.73 (1.40)** |
| **Influenza, seasonal (Flulaval)** | **72** | **8.04 (6.37–10.15)** | **8.00 (436.87)** | **7.93 (6.53)** | **2.99 (2.65)** |
| **Influenza A(H1N1) monovalent (Sanofi)** | **67** | **5.55 (4.36–7.06)** | **5.53 (246.73)** | **5.49 (4.49)** | **2.46 (2.10)** |
| **Influenza, seasonal (Afluria)** | **62** | **3.43 (2.67–4.40)** | **3.42 (105.39)** | **3.40 (2.76)** | **1.77 (1.40)** |
| **Zoster (unspecified brand)** | **55** | **3.01 (2.31–3.93)** | **3.01 (73.24)** | **2.99 (2.40)** | **1.58 (1.19)** |
| **Influenza, seasonal (Flucelvax Quadrivalent)** | **45** | **2.73 (2.03–3.66)** | **2.72 (48.87)** | **2.71 (2.12)** | **1.44 (1.01)** |
| **Influenza, seasonal (Afluria Quadrivalent)** | **43** | **3.34 (2.48–4.51)** | **3.34 (69.97)** | **3.32 (2.58)** | **1.73 (1.30)** |
| **Influenza, seasonal (FluMist)** | **43** | **3.48 (2.58–4.70)** | **3.47 (75.31)** | **3.46 (2.69)** | **1.79 (1.35)** |
| **Meningococcal conjugate (Menactra)** | **43** | **3.39 (2.51–4.57)** | **3.38 (71.67)** | **3.37 (2.62)** | **1.75 (1.31)** |
| **Influenza, seasonal (Fluad)** | **38** | **4.87 (3.54–6.70)** | **4.85 (115.75)** | **4.83 (3.70)** | **2.27 (1.81)** |
| **Hepatitis A + B (Twinrix)** | **37** | **2.99 (2.16–4.13)** | **2.98 (48.61)** | **2.97 (2.27)** | **1.57 (1.10)** |
| **RSV (Abrysvo)** | **37** | **6.82 (4.94–9.43)** | **6.79 (182.00)** | **6.76 (5.16)** | **2.76 (2.29)** |
| **RSV (Arexvy)** | **30** | **3.01 (2.10–4.30)** | **3.00 (39.91)** | **2.99 (2.22)** | **1.58 (1.06)** |
| **Influenza A(H1N1) monovalent (Novartis)** | **28** | **3.42 (2.36–4.96)** | **3.42 (47.72)** | **3.41 (2.50)** | **1.77 (1.23)** |
| **Influenza, seasonal (foreign/unspecified)** | **19** | **4.54 (2.89–7.12)** | **4.52 (52.06)** | **4.51 (3.10)** | **2.17 (1.53)** |
| **Influenza, seasonal (TIV, Dresden)** | **18** | **4.75 (2.99–7.55)** | **4.74 (53.02)** | **4.73 (3.21)** | **2.24 (1.58)** |
| **Rabies (Rabipur)** | **13** | **5.97 (3.46–10.29)** | **5.94 (53.41)** | **5.94 (3.76)** | **2.57 (1.80)** |
| **Influenza A(H1N1) monovalent (CSL)** | **12** | **7.05 (4.00–12.44)** | **7.02 (61.89)** | **7.01 (4.36)** | **2.81 (2.01)** |
| **Influenza, seasonal (QIV, Dresden)** | **8** | **5.23 (2.61–10.47)** | **5.21 (27.20)** | **5.20 (2.91)** | **2.38 (1.42)** |

Vaccines are those meeting all four signal thresholds simultaneously and are ordered by the number of GBS reports. Signal thresholds: ROR, lower limit of the 95% CI >1 with ≥3 reports; PRR ≥2 with χ² ≥4 and ≥3 reports; IC_025_ >0; EBGM_05_ >2. Values in parentheses are the 95% CI for ROR, the χ² statistic for PRR, and the lower bounds (EBGM_05_, IC_025_) for EBGM and IC. Abbreviations: CI, confidence interval; EBGM, empirical Bayesian geometric mean; EBGM_05_, lower fifth percentile of EBGM; GBS, Guillain-Barré syndrome; IC, information component; IC_025_, lower limit of the 95% credibility interval of IC; n, number of GBS reports; PRR, proportional reporting ratio; ROR, reporting odds ratio; RSV, respiratory syncytial virus; Tdap, tetanus–diphtheria–acellular pertussis; VAERS, Vaccine Adverse Event Reporting System; χ², chi-squared statistic.
